# Supplementary material for: Fermi surface and effective masses in photoemission response of the (Ba1−xKx)Fe2As2 superconductor
Source: Sci Rep. 2017 Aug 18;7:8787. doi: 10.1038/s41598-017-09480-y (PMC5562888; doi:10.1038/s41598-017-09480-y)
Supplement: Supplementary file 1 — Supplemental Material [file 41598_2017_9480_MOESM1_ESM.pdf]

# Supplemental Material for

### Fermi surface and effective masses in photoemission response of the $(Ba_{1-x}K_x)Fe_2As_2$ superconductor

Gerald Derondeau,[1,*] Federico Bisti,[2] Masaki Kobayashi,[2,3] Jürgen Braun,[1] Hubert Ebert,[1] Victor A. Rogalev,[2] Ming Shi,[2] Thorsten Schmitt,[2] Junzhang Ma,[2,4,5] Hong Ding,[4,5] Vladimir N. Strocov,[2,†] and Ján Minár[6,‡]

[1]*Department Chemie, Physikalische Chemie, Universität München, Butenandtstr. 5-13, 81377 München, Germany*

[2]*Swiss Light Source, Paul Scherrer Institute, CH-5232 Villigen PSI, Switzerland*

[3]*Department of Applied Chemistry, School of Engineering, University of Tokyo, 7-3-1 Hongo, Bunkyo-ku, Tokyo 113-8656, Japan*

[4]*Beijing National Laboratory for Condensed Matter Physics*

[5]*Institute of Physics, Chinese Academy of Sciences, Beijing 100190, China*

[6]*NewTechnologies-Research Center, University of West Bohemia, Pilsen, Czech Republic*

(Dated: June 7, 2017)

## 1. LIFSHITZ TRANSITION

A Lifshitz transition is characterized as a topological change of the Fermi surface.[1] For the iron pnictide superconductors this type of transition is of crucial importance as it is believed to mark the onset of superconductivity.[2–5] The K-doped $(Ba_{1-x}K_x)Fe_2As_2$ is a famous example for such a Lifshitz transition around the X point, leading to the discussed propeller topologies seen in ARPES experiments.[5–8] As known from experimental data [6–8] and the experiments performed within this work these topological features are already clearly visible for the optimally doped $(Ba_{0.6}K_{0.4})Fe_2As_2$. This is expected, following the argumentation that the Lifshitz transition suppresses the magnetic order due to a reduced nesting and thus induces superconductivity.[2, 9] However, the observed Lifshitz transition on the basis of the LDA was so far discussed only for high doping concentrations $x \approx 0.9$. [5, 10] It is also known that it is difficult to prepare homogeneous samples of over-doped $(Ba_{1-x}K_x)Fe_2As_2$ [5] which might explain discrepancies between various experiments about the onset of the Lifshitz transition.[6, 8, 10, 11] One remarkable and important paper from Khan and Johnson used the CPA to investigate the Lifshitz transition for $(Ba_{1-x}K_x)Fe_2As_2$ and they found a similar emergence of these propeller-like topologies for the heavily over-doped $(Ba_{0.1}K_{0.9})Fe_2As_2$.[5] However, with our new findings this result is now fully understandable. Using only a LDA based approach the relevant bands around X are still around 0.1 eV below the Fermi level ($E_F$). One can use over-doping with K in order to decrease $E_F$. The disadvantage of this approach is however that not only the relevant bands around X but the whole band structure is moved. Using a LDA+DMFT based approach one can see that correlation effects alter the electronic structure around X already for optimally doped $(Ba_{0.6}K_{0.4})Fe_2As_2$ so that the Lifshitz transition can emerge for lower doping concentration, in agreement with experiments.[6–8]

In order to show how this topology is affected by the correlation strength we show the corresponding BSF and FS in Fig. S1 for (A) LDA and LDA+DMFT with a varying on-site Coulomb interaction of (B) $U = 2.0$ eV, (C) $U = 3.0$ eV and (D) $U = 4.0$ eV with a constant exchange interaction $J = 0.9$ eV for Fe. Best agreement with experiment can be found for $U = 3.0$ eV as used and discussed in the main paper but it is also obvious that bands responsible for the Lifshitz transition are directly controlled by the Coulomb interaction $U$. Thus, we show that the origin of the important Lifshitz transition in $(Ba_{1-x}K_x)Fe_2As_2$ can be fully explained by correlation effects.

Still, it is also interesting to note that recent work on the ARPES spectra of the electron doped $Ba(Fe_{1-x}Co_x)_2As_2$ was successful using only a LDA approach.[12] The strength of correlation effects in the iron pnictides seem to vary with electron or hole doping.

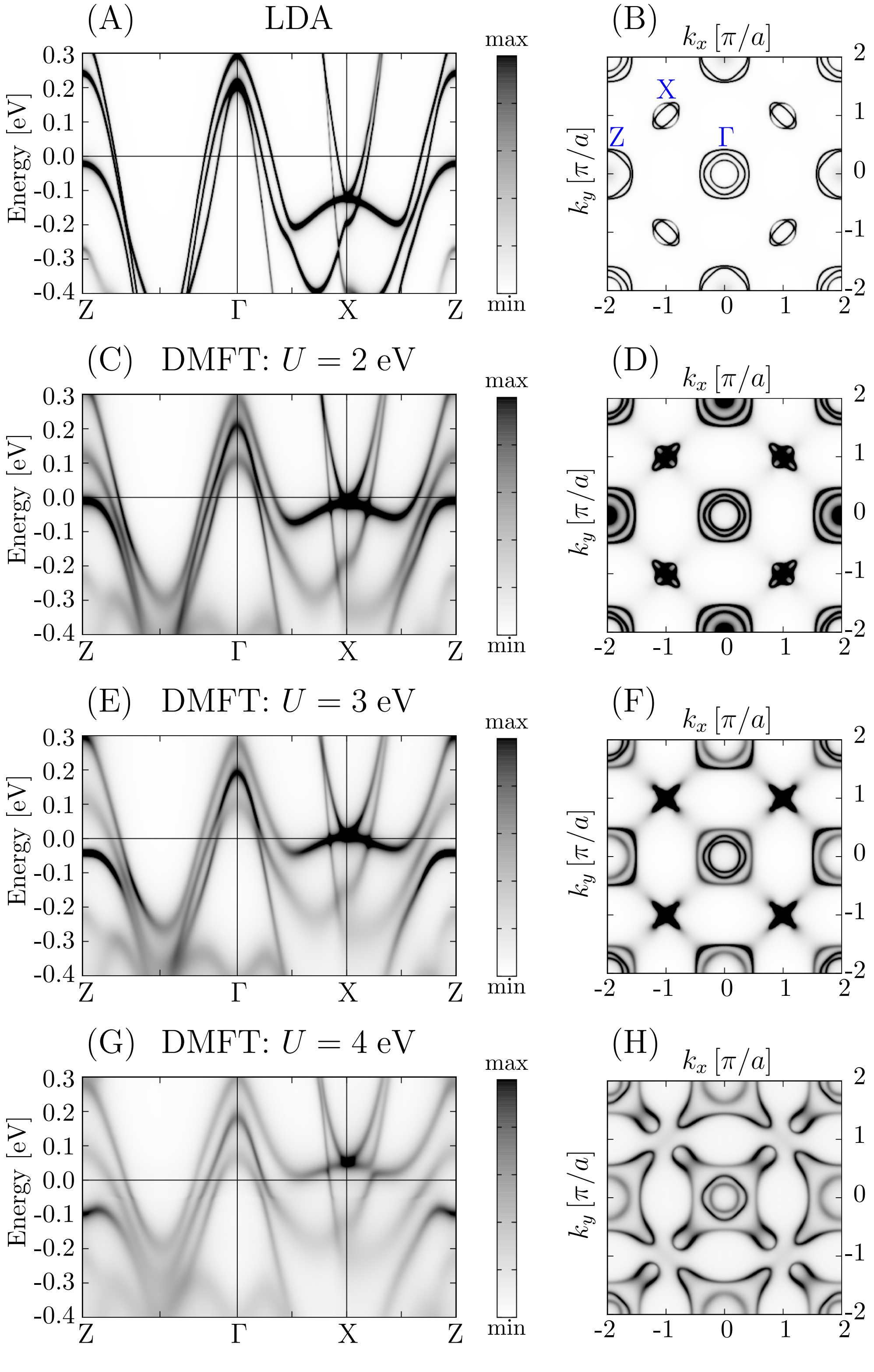


FIG. S1. (Color online) (A + B) BSF and FS of $(Ba_{0.6}K_{0.4})Fe_2As_2$ calculated on the basis of LDA. Corresponding BSF and FS of $(Ba_{0.6}K_{0.4})Fe_2As_2$ calculated on the basis of LDA+DMFT with (C + D) $U = 2.0$ eV, (E + F) $U = 3.0$ eV and (G + H) $U = 4.0$ eV for Fe ($J = 0.9$ eV).

## 2. EXTENDED FERMI SURFACE CUT

In correspondence to Fig. 3 of the main manuscript we present additionally extended Fermi surface cuts of $(Ba_{0.6}K_{0.4})Fe_2As_2$ for experimental data and theoretical calculations, respectively. Thus, Fig. S2 (A) shows the experimental data up to the second Brillouin zone and (B) the calculated Fermi surface cut up to the forth Brillouin zone, both images for $p$-polarized light and $h\nu = 430$ eV. This verifies that the alternating symmetry of the flower-like topology at $\bar{\Gamma}'$ compared to $\bar{\Gamma}$ is preserved over the whole $k$-space, in agreement with the data of Zabolotnyy *et al.* [6]. Interestingly, the flower-like topology changes a little bit for the third and forth Brillouin zone in Fig. S2 (B) which is connected to the $k_z$ dispersion. Note, that the calculation is shown for a fixed photon energy of $h\nu = 430$ eV, thus, the effect of the $k_z$ dispersion increases for higher Brillouin zones (see also the $k_z$ scan in Fig. 4). In the experimental data of Fig. S2 (A) the value of $k_z$ is corrected on the other hand. The blue square in (B) corresponds to the part of the Fermi surface shown in (A).

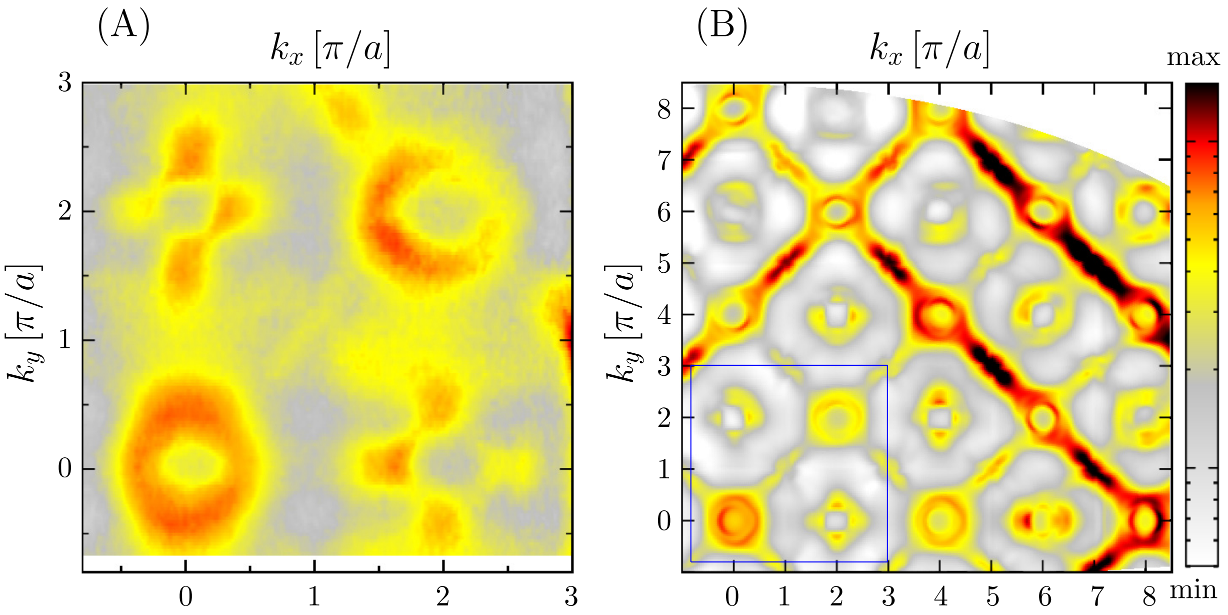


FIG. S2. Fermi surface cuts of $(Ba_{0.6}K_{0.4})Fe_2As_2$ with a fixed $h\nu = 430$ eV and for $p$-polarized light extending over several Brillouin zones. This is shown for (A) experimental data and (B) ARPES calculations using LDA+DMFT. The blue square in (B) corresponds to the part seen in (A).

## 3. DERIVED EFFECTIVE MASS ENHANCEMENT

In order to derive the mass enhancements shown in Tab. I the corresponding bands shown in Fig. S3 were used with the applied parabolic fits shown as solid green and red lines on top. For the BSF in Fig. S3 (A + B) and (E + F) the band dispersion can be fitted very reasonably by parabolic

functions. For the ARPES response however, final state effects, matrix element effects and surface effects alter the true band dispersion which results in bands showing more deviation from the perfect parabolic behavior. As discussed in the main paper, this is mainly due to final state effects which have a stronger impact the more pronounced the $k_z$ dispersion of the corresponding bands is. Thus, the deviations from the perfect parabolic behavior are stronger in Fig. S3 (C + D) at the Γ point, compared to Fig. S3 (G + H) at the X point, because the bands at Γ have stronger 3D character whereas the band at X considered here is almost a 2D band. Furthermore, one should note that in the ARPES spectra the two outer bands around Γ are hardly distinguishable due to intensity loss connected to the ARPES response. As a technical detail, the calculations used an imaginary energy of 0.025 eV for the initial states and of 5.0 eV for the final states.

The resulting mass renormalization is shown in Fig. S4 (A - C) with the LDA bands from Fig. S3 (A) on top of the respective DMFT BSF and the DMFT ARPES response as dashed green and red lines. In addition, the DMFT bands of Fig. S3 (B) are shown on top of the ARPES spectra in Fig. S4 (D - E). This shows explicitly the deviation of the ARPES response from the true band dispersion derived from DMFT BSF. Consequently, the apparent mass enhancement seen in

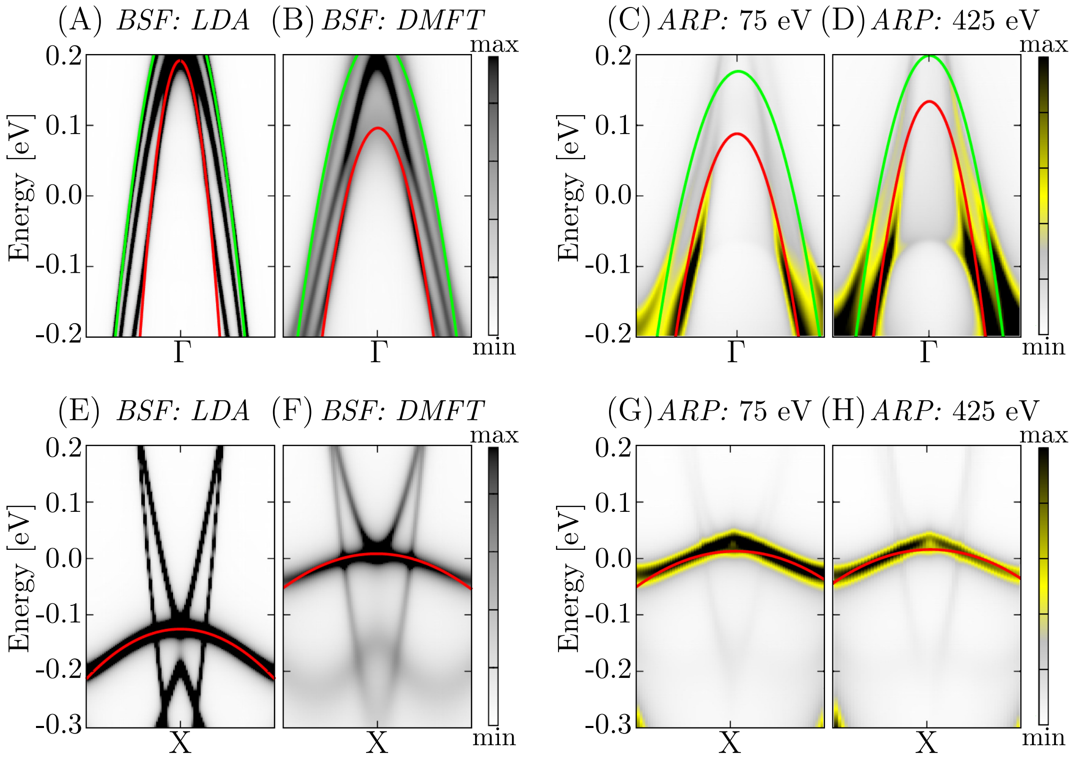


FIG. S3. Bands for which the mass enhancement is shown in Tab I. The solid red and green lines correspond to the applied parabolic plot fit. They are shown for (A - D) the Γ point having a strong $k_z$ dispersion and for (E - H) the X point having a weak $k_z$ dispersion. (A + E) BSF based on LDA, (B + F) BSF based on LDA+DMFT and (C + G) ARPES calculations based on DMFT for $h\nu = 75$ eV and (D + H) the same for $h\nu = 425$ eV.

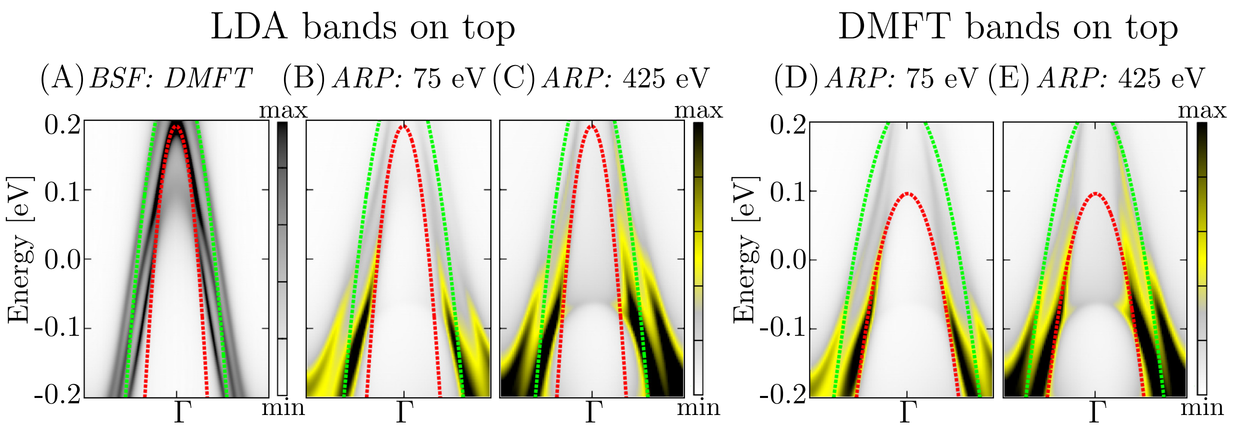


FIG. S4. (A - C) Band renormalization for BSF DMFT and the ARPES response with the LDA bands of Fig. S3 (A) on top. (D - E) The same ARPES spectra but with the DMFT bands of Fig. S3 (B) on top in order to show the effect only due to the ARPES response.

ARPES is affected by these ARPES response effects and thus it is not an intrinsic property of the quasiparticle valence band structure or spectral function. Why the ARPES response is affected by the $k_z$ broadening $\Delta k_z$ is depicted schematically in Fig. S5. As discussed in the main paper, $\Delta k_z$ can be reduced by choosing a higher photon energy, although the resulting effects can be never fully avoided for 3D materials like the iron pnictides.

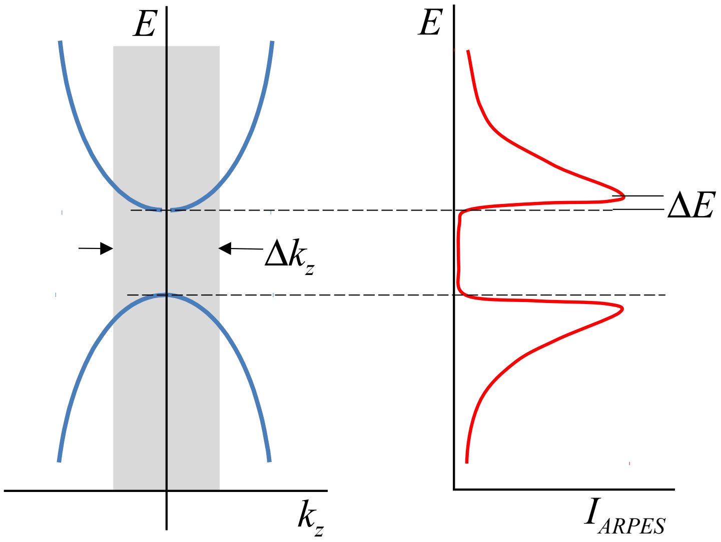


FIG. S5. Mechanism of the apparent mass enhancement (assuming a constant matrix element). The ARPES signal is formed by averaging of the valence band dispersion within the $\Delta k_z$ broadening interval. Near the extremes of the $k_z$ dispersion, this averaging results in asymmetry of the ARPES weight and shifting of the resulting spectral peak away from the extreme towards the band interior (for a more detailed picture including lifetime broadening of the valence states see Ref. [13]). Concerning its $\boldsymbol{k}_{||}$ dependence of the ARPES spectra this shift causes an apparent reduction of the bandwidth and an increase of $m^*$.

* gerald.derondeau@cup.uni-muenchen.de
† vladimir.strocov@psi.ch
‡ jminar@ntc.zcu.cz

[1] I. M. Lifshitz, Sov. Phys. J.E.T.P. **11**, 1130 (1960).

[2] C. Liu, T. Kondo, R. M. Fernandes, A. D. Palczewski, E. D. Mun, N. Ni, A. N. Thaler, A. Bostwick, E. Rotenberg, J. Schmalian, S. L. Bud'ko, P. C. Canfield, and A. Kaminski, Nature Physics **6**, 419 (2010).

[3] K. Matan, S. Ibuka, R. Morinaga, S. Chi, J. W. Lynn, A. D. Christianson, M. D. Lumsden, and T. J. Sato, Phys. Rev. B **82**, 054515 (2010).

[4] C. Liu, A. D. Palczewski, R. S. Dhaka, T. Kondo, R. M. Fernandes, E. D. Mun, H. Hodovanets, A. N. Thaler, J. Schmalian, S. L. Bud'ko, P. C. Canfield, and A. Kaminski, Phys. Rev. B **84**, 020509 (2011).

[5] S. N. Khan and D. D. Johnson, Phys. Rev. Lett. **112**, 156401 (2014).

[6] V. B. Zabolotnyy, D. S. Inosov, D. V. Evtushinsky, A. Koitzsch, A. A. Kordyuk, G. L. Sun, J. T. Park, D. Haug, V. Hinkov, A. V. Boris, C. T. Lin, M. Knupfer, A. N. Yaresko, B. Büchner, A. Varykhalov, R. Follath, and S. V. Borisenko, Nature **457**, 569 (2009).

[7] D. V. Evtushinsky, D. S. Inosov, V. B. Zabolotnyy, M. S. Viazovska, R. Khasanov, A. Amato, H.-H. Klauss, H. Luetkens, C. Niedermayer, G. L. Sun, V. Hinkov, C. T. Lin, A. Varykhalov, A. Koitzsch, M. Knupfer, B. Büchner, A. A. Kordyuk, and S. V. Borisenko, New Journal of Physics **11**, 055069 (2009).

[8] D. V. Evtushinsky, A. A. Kordyuk, V. B. Zabolotnyy, D. S. Inosov, T. K. Kim, B. Büchner, H. Luo, Z. Wang, H.-H. Wen, G. Sun, C. Lin, and S. V. Borisenko, J. Phys. Soc. Japan **80**, 023710 (2011).

[9] M. G. Kim, J. Lamsal, T. W. Heitmann, G. S. Tucker, D. K. Pratt, S. N. Khan, Y. B. Lee, A. Alam, A. Thaler, N. Ni, S. Ran, S. L. Bud'ko, K. J. Marty, M. D. Lumsden, P. C. Canfield, B. N. Harmon, D. D. Johnson, A. Kreyssig, R. J. McQueeney, and A. I. Goldman, Phys. Rev. Lett. **109**, 167003 (2012).

[10] N. Xu, P. Richard, X. Shi, A. van Roekeghem, T. Qian, E. Razzoli, E. Rienks, G.-F. Chen, E. Ieki, K. Nakayama, T. Sato, T. Takahashi, M. Shi, and H. Ding, Phys. Rev. B **88**, 220508 (2013).

[11] K. Nakayama, T. Sato, P. Richard, Y.-M. Xu, T. Kawahara, K. Umezawa, T. Qian, M. Neupane, G. F. Chen, H. Ding, and T. Takahashi, Phys. Rev. B **83**, 020501 (2011).

[12] G. Derondeau, J. Braun, H. Ebert, and J. Minár, Phys. Rev. B **93**, 144513 (2016).

[13] V. N. Strocov, J. Electron. Spectrosc. Relat. Phenom. **130**, 65 (2003).
